# Menaquinone-7 Supplementation Improves Osteogenesis in Pluripotent Stem Cell Derived Mesenchymal Stem Cells

**DOI:** 10.3389/fcell.2020.618760

**Published:** 2021-01-28

**Authors:** Asim Cengiz Akbulut, Grzegorz B. Wasilewski, Nikolas Rapp, Francesco Forin, Heike Singer, Katrin J. Czogalla-Nitsche, Leon J. Schurgers

**Affiliations:** ^1^Department of Biochemistry, Cardiovascular Research Institute Maastricht, Maastricht University, Maastricht, Netherlands; ^2^NattoPharma ASA, Oslo, Norway; ^3^Institute of Experimental Haematology and Transfusion Medicine, University Clinic Bonn, Bonn, Germany; ^4^Department of Nephro-Cardiology, Rheinisch-Westfälische Technische Hochschule Klinikum, Aachen, Germany

**Keywords:** vitamin K, menaquinone-7, osteogenesis, pluripotent stem cells, mesenchymal stem cells

## Abstract

Development of clinical stem cell interventions are hampered by immature cell progeny under current protocols. Human mesenchymal stem cells (hMSCs) are characterized by their ability to self-renew and differentiate into multiple lineages. Generating hMSCs from pluripotent stem cells (iPSCs) is an attractive avenue for cost-efficient and scalable production of cellular material. In this study we generate mature osteoblasts from iPSCs using a stable expandable MSC intermediate, refining established protocols. We investigated the timeframe and phenotype of cells under osteogenic conditions as well as the effect of menaquinone-7 (MK-7) on differentiation. From day 2 we noted a significant increase in RUNX2 expression under osteogenic conditions with MK-7, as well as decreases in ROS species production, increased cellular migration and changes to dynamics of collagen deposition when compared to differentiated cells that were not treated with MK-7. At day 21 OsteoMK-7 increased alkaline phosphatase activity and collagen deposition, as well as downregulated RUNX2 expression, suggesting to a mature cellular phenotype. Throughout we note no changes to expression of osteocalcin suggesting a non-canonical function of MK-7 in osteoblast differentiation. Together our data provide further mechanistic insight between basic and clinical studies on extrahepatic activity of MK-7. Our findings show that MK-7 promotes osteoblast maturation thereby increasing osteogenic differentiation.

## Introduction

Mesenchymal stem cells (MSCs) are the predominant source of cells for endogenous repair after bone fracture (Wang et al., 2013). Application of MSCs in regenerative medicine has been widespread and explored as a therapeutic source following damage to bone, cartilage, cardiac, tendon, and immune-related disorders (Wang et al., 2020). The ability to more specifically program and direct cells beforehand could improve regenerative outcome (Levy et al., 2020; Tsai et al., 2020; Varkouhi et al., 2020). Induced pluripotent stem cells (iPSCs) allow for an unlimited supply of patient specific cells. Furthermore, the establishment of clinically validated lines could streamline the process of applying regenerative cell-based therapies to the clinic (Sullivan et al., 2018). Current protocols are scarce in variety of chemicals that are used for differentiating bone, mainly consisting of Dexamethasone, Beta-Glycerol Phosphate and L-Ascorbic Acid (Vitamin C) (Ciuffreda et al., 2016). It is clear that such limited number of stimuli cannot replace physiological bone formation and are prone to achieve suboptimal osteogenesis conditions, thereby contributing to variability in these processes (Nweke and Stegemann, 2020). Additional supplementation to mineralisation media might aid the *in vitro* development of bone and could be beneficial to enhancing the expression profile of bone-related markers.

Vitamin K was first discovered in the 1930s for its role in coagulation, and the extrahepatic activity of vitamin K has been widely unexplored (Dam and Schonheyder, 1936). Vitamin K is a hypernym for multiple analogs each with their own distinct properties and structure. The past decades have begun to elucidate a potent role for vitamin K2 in health and disease beyond coagulation (Halder et al., 2019). Vitamin K2 analog menaquinone-7 (MK-7) has been implicated in a range of studies, from basic biochemistry to long term clinical studies (Iwamoto et al., 2009; Apalset et al., 2011; Bulló et al., 2011; Evatt et al., 2011). The majority of these studies have implicated an important role for MK-7 in bone health and metabolism, yet studies on the mechanistic role of MK-7 on bone healing are lacking (Wasilewski et al., 2019; Akbulut et al., 2020).

Using iPSCs to generate MSCs is the next step in combining the best from stem cell technology and tissue engineering (Zhao and Ikeya, 2018). Further, differentiating cells from pluripotency toward mesenchymal and osteogenic lineage enables application of a novel tool for interrogation of developmental pathways and phenotypes in bone formation (Yousefi et al., 2016). This can potentially answer age-old questions with regards to mechanisms of endochondral transdifferentiation, and the role of macrophages in bone formation (Setty, 2014; Cho, 2015; Dogan, 2018; Haraguchi et al., 2019; Marín-Llera et al., 2019). Present methods of determination of osteogenic processes include detection of osteoblastic markers such as RUNX2, Col1A1, and Osterix along with ALP activity and calcium deposition (Svandova et al., 2020; Yong et al., 2020). However, oxidative stress status or cell migration are rarely explored and should be delineated to enrich the knowledge of differentiation and repair processes in osteogenesis (Kubo et al., 2019).

Due to the aforementioned obstacles to clinical application of stem cell therapies, we investigated the role of vitamin K analog menaquinone-7 [MK-7 (VK2)], in the differentiation of iPSCs to osteoblasts using a stable iMSC intermediate. The intermediate phase allows for reduced costs and technical demands, developing an application toward widescale of tissue engineering based solutions. In this study we find that supplementation of MK-7 to osteogenic medium promotes differentiation of iMSCs toward osteoblasts significant beyond that of osteogenic medium alone.

## Materials and Methods

### Generation and Maintenance of Pluripotent Stem Cell Line

The iPSC line iPSC-UkB-Ctrl-XX was generated from PBMCs *via* expansion to a cellular intermediate of EPCs, using Stem Span SFEM II (Stem Cell Technologies) and addition of Erythropoietin (R&D Systems), Stem Cell Factor (PeproTech), Interleukin-3 (PeproTech), Insulin Growth Factor-1 (PeproTech), and dexamethasone (Sigma-Aldrich). Following expansion, EPCs were electroporated with Epi5 Reprogramming (Thermo Fisher Scientific) Lonza 4D-Nucleofector X-Unit (Lonza). Cells were plated on Corning ESC-grade Matrigel (Corning) in ReproTeSR (Stem Cell Technologies) before changing to mTeSR 1 (Stem Cell Technologies) when colonies first appeared. Following iPSC clones were picked and expanded before characterization. iPSC-UkB-Ctrl-XX line is cultured on Corning ESC-grade Matrigel and in mTeSR 1. Cells are passaged when colonies are too large using 0.5 mM EDTA in PBS and cultured in a humidified incubator at 37°C and 5% CO_2_.

### Generation and Maintenance of iMSCs

iMSCs were generated as previously described (Kang et al., 2015). Briefly, cell line iPSC-UkB-Ctrl-XX was passaged as normal the day before starting differentiation. Differentiation medium was added and refreshed every 2 days for 2 weeks. iMSC differentiation medium contains Dulbecco's Modified Eagle Medium (DMEM) low glucose (1 g/L D-Glucose), 10% Fetal Bovine Serum (FBS), 1% Penicillin/Streptomycin (Pen/Strep), 2 mM glutamine and 0.05 mM L-ascorbic acid. Following the initial 2 weeks, iMSCs were passaged with a split ratio of 1:2 on to 0.1% gelatine-coated 6-well plates for two further passages before adhering to untreated plastic. At this stage, cells assume morphology similar to that of primary MSCs and were maintained in differentiation medium with a split ratio of either 1:2 or 1:3. Cells were cultured in a humidified incubator at 37°C and 5% CO_2_.

### Osteogenic Differentiation of iMSCs

Osteogenic differentiated iMSC were used between 8 and 20 passages. iMSCs were seeded on appropriate size plates dependent on analysis with two varying densities depending on time in culture. For earlier timepoints (2, 7, and 10 days), cells were seeded at 10^5^ cells per cm^2^ whereas for the later time point (21 days), cells were seeded at 2.6 × 10^4^ per cm^2^. The following day, medium was changed to osteogenic medium composed of DMEM, FBS (10%), Pen/Strep (1%), 100 nM Dexamethasone, 10 mm B-Glycerol—phosphate, and 0.05 mM L-ascorbic acid. For MK-7 (synthetic menaquinone-7, kind gift NattoPharma, Oslo, Norway) supplementation a final concentration of 10 μM was added or equal volume of isopropanol vehicle control.

### Chondrogenic Differentiation of iMSCs

Differentiation medium was as follows; high glucose DMEM, TGF-B1 10 ng ml^−1^ (PeproTech), 100 nM dexamethasone (Sigma-Aldrich), insulin (Sigma-Aldrich). Cells were seeded at a density of 2.6 × 10^4^ per cm^2^. Medium was refreshed three times per week and cells lysed after 2 weeks of differentiation induction. Control cells were cultured in standard medium with appropriate vehicle controls.

### Adipogenic Differentiation of iMSCs

Adipogenic differentiation was induced by seeding cells at 2 × 10^4^ cell per cm^2^. Differentiation medium was composed of high glucose DMEM, FBS (10%), Pen-Strep (1%), 0.5 mM isobutylmethylxanthine and 1 mM dexamethasone. Medium was refreshed every 2 or 3 days and cells were lysed for analysis after 2 weeks of differentiation. The control cells were cultured in standard medium with appropriate vehicle controls.

### Vasculogenic Differentiation of iMSCs

Differentiating iMSCs to vascular smooth muscle-like cells was done using vasculogenic differentiation medium composed of high glucose DMEM, FBS (10%), Pen-Strep (1%), PDGF-BB 10 ng ml^−1^ (PeproTech) and TGF-B1 5 ng ml^−1^ (PeproTech). Cells were seeded at a density of 2 × 10^4^ cells per cm^2^. Medium was refreshed every 2–3 days for 14 days before analysis. Control cells were cultured in standard medium with appropriate vehicle controls.

### Real-Time Reverse-Transcription Polymerase Chain Reaction

Total RNA was isolated from differentiated cells using TRIzol reagent (Invitrogen) and quantified using a NanoDrop 2000 spectrophotometer (Thermo Fisher Scientific). Five hundred nanogram of total RNA was transcribed using iScript^TM^ Reverse Transcription Supermix (BioRad) for RT-qPCR in a 20 μl reaction. The resulting cDNA was diluted one and a half times, and 4 mL was amplified using Power SYBR Green PCR System following manufacturers recommendations. Real-time qPCR was performed using the Quantitect SYBR green PCR kit (Qiagen) in a LightCycler 480 II (Roche) with 50 ng of cDNA and 0.5 μM of each primer. The sequences of primers used are provided in Supplementary Table 1. Relative expression for each gene was normalized against GAPDH and expressed as fold change over control. Fluorescence curves were analyzed with LightCycler 480 Software (Version 1.5) and relative quantification was performed with the 2^−ΔΔCt^ method. Data from at least 3 different differentiations of our line were combined and reported as mean ± SD.

### Flow Cytometric Analysis

iMSCs were dissociated, washed and adjusted to a cell suspension of concentration 1 × 10^6^ in ice-cold PBS, 10% FBS and 1% sodium azide at 4°C. Next, the conjugated primary antibody was added in 3% FBS in PBS for 30 min in dark at room temperature. After the incubation, cells were washed 3x by centrifugation at 400 g for 5 min and resuspended in 1 ml ice-cold PBS, 10% FCS, 1% sodium azide. Cells were kept at ice until time of analysis.

### O-Cresolphthalein Assay

Cells in 48 well plates were washed twice with PBS. The mineralized matrix was dissolved in 1 M HCl and put on a shaker overnight. To quantify the amount of calcium per sample, Randox O-cresolphthalein kit was used to assess the amount of calcium embedded in extracellular matrix. Values were converted to μg calcium and adjusted to protein levels. All samples were assayed in triplicate.

### DC Protein Assay

For normalization of the calcium content of the cells, DC protein assay was performed. 1 M HCl cell suspension was neutralized and lysed with 1 M NaOH 0.2% SDS and incubated on a shaker overnight. Plates were read at 750 nm using Cytation3 (BioTek). Standard curve was created and sample absorbances were calculated, giving the protein content μg/μl protein. All samples were assayed in triplicate in three independent experiments.

### Immunofluorescence

Cells were fixed with 4% PFA, permeabilized with 0.1% Triton X-100, blocked in 1% BSA/PBS and incubated with primary antibodies (COL1a1, Applied Logistics). The following secondary antibodies were used: anti-rabbit-FITC (Dako, F0205) and anti-mouse-FITC (Dako, F0232). Nuclei were stained with DAPI (Sigma–Aldrich). Cells were analyzed using Cytation3.

### Western Blotting

VSMCs were lysed in 0.1 M Tris pH 8.1, 0.15 M NaCl, 1% triton x-100 0.2 mM NaVO_3_ and 1:50 protease inhibitor cocktail (Sigma). Protein concentration was determined using DC protein assay (Bio-Rad) and lysates were separated on Any kD Mini-PROTEAN TGX Precast Protein Gels (BioRad). Samples were transferred to nitrocellulose membrane (BioRad) and incubated overnight with anti-Runx2 (MBL, D130-3) and anti-Col1A1 (BD, 610153). Protein was detected using HRP-conjugated secondary antibodies (anti-mouse: p0447, Dako; anti-rabbit: 7074S, Cell Signaling, anti-goat: P0449, Dako) and visualized by enhanced chemiluminescence (Pierce ECL Western Blotting Substrate, ThermoFisher Scientific). All samples were blotted in triplicate.

### Alkaline Phosphatase Activity

Cells were lysed in 1% Triton X-100 in PBS, subjected to 2 freeze-thaw cycles and centrifuged at 13,000 g for 5min. ALP activity in the supernatants was measured at 405 nm using 4-Nitrophenyl phosphate disodium salt hexahydrate (Sigma) as substrate. Enzyme activity (U) was normalized to protein concentrations. All samples were assayed in triplicate.

### Alizarin Red Assay

On the final day of differentiation, medium was aspirated, and wells were washed twice with PBS. Next, cells were fixed in 4% formaldehyde in RT for 40 min. Then cells were washed twice with PBS and stained with 2% Alizarin Red for 1 h, followed by washing cells twice with PBS. Mineral deposits were visualized using light microscopy at 20X magnification.

### Reactive Oxygen Species

To measure oxidative stress, we measured reactive oxygen species (ROS) using 2, 7-dichlorofluoroscein diacetate (DCFDA, Merck) which is oxidized to 2, 7-dichlorofluoroscein in the presence of the oxidants. Mesenchymal stem cells supplemented with osteogenic media with or without MK-7 (10 μM final concentration) and left for 48 h, or 7 days with medium refreshed twice. After 2 or 7 days, media was replaced with Krebs-Ringer Phosphate Glucose Buffer (KRPG) in the presence of 20 μM DCFDA in the dark at 37°C and 5% CO_2_. Next, the fluorescence was measured (Excitation 485, Emission 529) with Cytation Cell Imaging Multi-Mode Reader (Bio-Tek Instruments) for a total of 45 min. Fluorescence intensity was normalized to the protein content.

### Migration Assay

Mesenchymal stem cells were seeded in cell culture plate until confluence was reached. Migration assay started at 0 h followed by scratching a monolayer of cells with a pipette tip. Cells were washed twice with PBS and supplemented with control and osteogenic media with or without MK-7 (10 μM final concentration) and left for 24 h. Gap closure was calculated in reference to timepoint = 0 using Image J [ImageJ 1.52q (64-bit)].

### xCELLigence Proliferation Assay

To monitor continuous real-time proliferation XCELLigence system was used Cells were seeded in microelectrode plate control and osteogenic media with or without MK-7 (10 μM final concentration) to measure impedance. Cell impedance were measured at 15 min intervals up to 168 h. Electrical impedance was a measurement of cell number and recorded as Cell Index (CI). Cells were kept in 37°C and 5% CO_2_ until the end of the experiment.

### Statistical Analysis

Results are presented as replicates in three or more independent experiments ± standard deviation. Statistical analysis was performed using GraphPad Prism (v8.4.3, Prism 8 for macOS, GraphPad Software, USA). Two-tailed unpaired Student's *t*-test was used for comparisons between two groups, or a one-way analysis of variance with a *post-hoc* test of Tukey's analysis when more than two groups were compared. Statistical significance between two groups that did not display normal distribution was performed using Mann-Whitney *U*-test. Statistical significance denoted by ^*^p < 0.05, ^**^p < 0.01, ^***^p < 0.001, ^****^p < 0.0001.

## Results

### Characterization of iPSC-UkB-Ctrl-XX and Generation of iMSC-Ctrl-1

**Figure 1:**
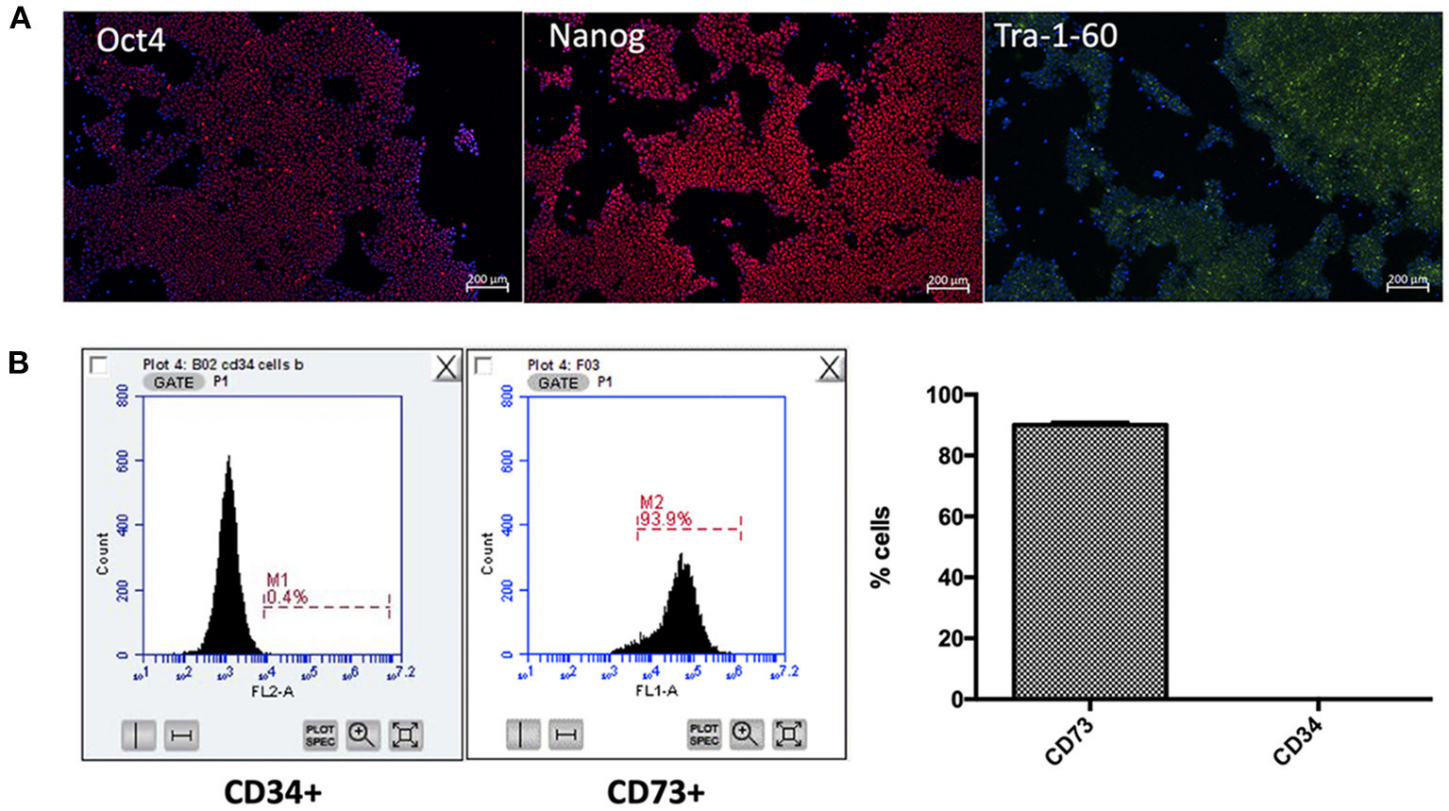
Characterization of iPSC-UkB-Ctrl-XX and generation of iMSC. Immunocytochemistry, **(A)** confirms pluripotency of cell line by expression of pluripotent markers Oct4, Nanog and Tra-1-60. **(B)** FACS sorting for CD73 and CD34 confirms that no cells are expressing hematopoietic stem cell marker CD34 and are positive for mesenchymal stem cell marker CD73.

Following generation of iPSC-UkB-Ctrl-XX, cell line was characterized by immunofluorescence for pluripotency markers (Figure 1A), trilineage differentiation (Ecto-/endo-/mesoderm) and karyotyped as normal (data not shown). Following differentiation to mesenchymal lineage, cells were FACS sorted to check for markers associated to mesenchymal and hematopoietic stem cells (Figure 1B). CD73 was used as marker for mesenchymal stem cell and cells highly positive (Lv et al., 2014), whereas CD34 used as a marker for hematopoietic stem cells showed negative (Ye et al., 2004). This gives us the confidence moving forward into osteogenic differentiations that what we are observing is a model for mesenchyme for osteoblast to osteocyte differentiation without possible mechanisms that involve osteoclasts.

### Differentiation Potential of iMSCs

**Figure 2:**
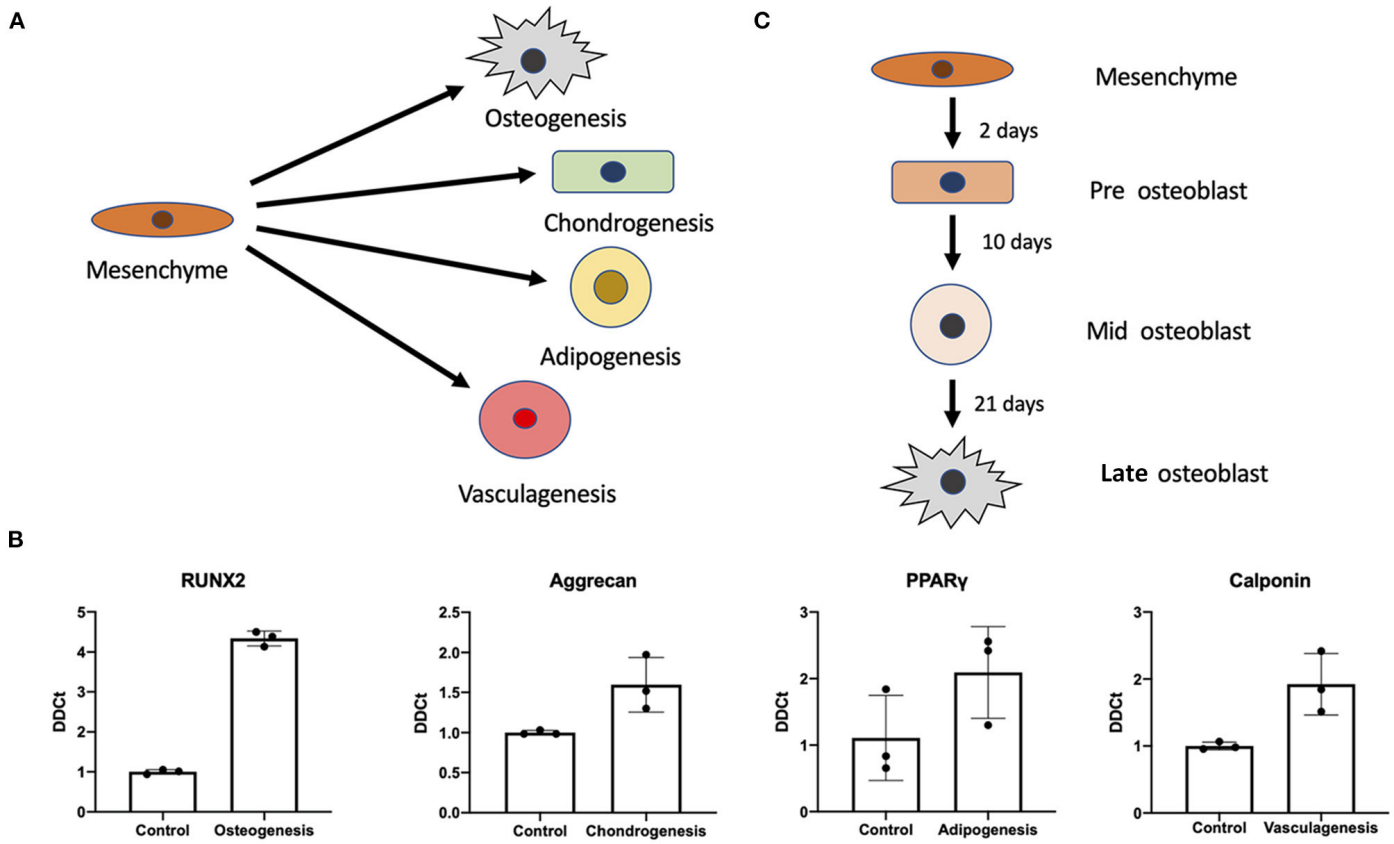
Mesenchymal stem cell differentiation potential. Differentiation potential of mesenchymal stem cells. **(A)** Visual representation of the different cellular iMSCs have potential to generate (osteogenic, chondrogenic, adipogenic, and vasculagenic lineages). RT-qPCR confirms differentiation of these four lineages by expression of commonly used markers RUNX2, aggrecan, PPARgamma, calponin (respectively) **(B)**. Variant time course of osteoblastic differentiation that was interrogated to check time points for pre-osteoblast, mid-osteoblast and mature osteoblast phenotype **(C)**.

Using previously established protocols for primary cells we tested whether differentiation of iMSCs was possible into the canonical triad of expected mesenchymal differentiations as well as vasculogenic potential (Figure 2A). We found that upon differentiation iMSCs responded successfully to stimuli as verified by RT-qPCR for markers synonymous with the various progeny (Figure 2B). This proof of efficacy provides us with a definitive cell source capable for performing further studies into mesenchymal stem cell differentiations.

### Variant Time Course of Osteogenic Differentiation

Next we interrogated the mesenchymal osteogenic trajectory at a series of timepoints of osteoblast differentiation (Figure 2C). We investigated expression of a variety of genes associated with bone formation under osteogenic treatment (Osteo), osteogenic treatment with MK-7 supplementation (OsteoMK-7) and the respective controls (control and MK-7). These conditions were used to interrogate osteoblast differentiation at various stages considered early, mid and late differentiation, days 2, 10, and 21, respectively (Figure 2C).

**Figure 3:**
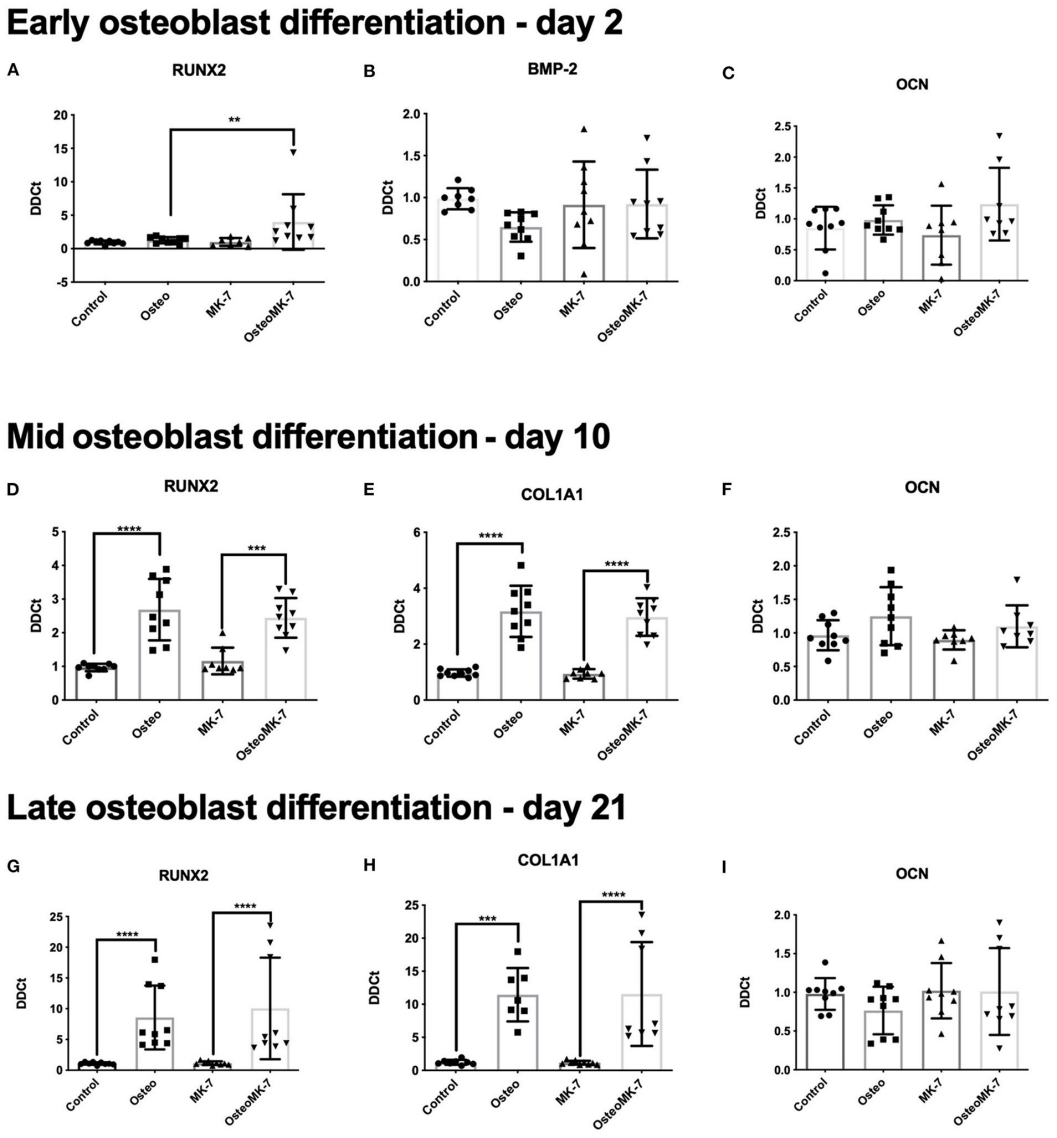
RT-qPCR expression of markers at various time points in differentiation. RT-qPCR of RUNX 2 **(A,D,G)** at various time points reveals its upregulation from controls as early as timepoint day 2 wherein RUNX2 is significantly upregulated under OsteoMK7 compared to controls and Osteo (*p* = 0.0040). At earliest timepoint BMP2 expression was not modulated between any treatments **(B)**. COL1A1 **(E,H)** is significantly upregulated in osteogenic differentiation from day 10 (*p* < 0.0001). However, osteocalcin **(C,F,I)** has no differences in expression *via* any of the treatment over any time point in the differentiation. **p < 0.01, ***p < 0.001, ****p < 0.0001.

Runt-related transcription factor 2 (RUNX2) is a master transcription factor for early osteogenic differentiation. We measured expression at earliest time point and found RUNX2 to be significantly upregulated in OsteoMK-7 treatment compared to Osteo and both controls (*p* < 0.005, Figure 3A). BMP-2 is suggested to play an important role in early bone formation and OCN (osteocalcin), is a vitamin K dependent protein (VKDP) present in mature osteoblasts. Therefore, we investigated whether our treatments change the expression of these markers. Expression of BMP-2 and OCN at day 2 did not vary between any treatments (Figures 3B,C). At mid-osteoblast differentiation (day 10), RUNX2 was further upregulated in osteogenic differentiation, although this was no longer in favor of OsteoMK-7 condition (Figure 3D). Further, at this timepoint we noticed an upregulation of COL1A1 expression under Osteo (*p* < 0.0001) and OsteoMK-7 (*p* < 0.0001) conditions, without favourability to OsteoMK-7 (Figure 3E). OCN expression did not differ in expression between any treatments at day 10 (Figure 3F). At late-osteoblast differentiation (day 21), both RUNX2 and COL1A1 have an even higher fold upregulation (~10-fold for both) compared to the controls then that of day 10 (Figures 3G,H). Also, at the late-osteoblast differentiation there is no difference in expression of either of the genes measured for OsteoMK-7 treatment. Furthermore, we did not observe any difference in OCN expression under any treatments compared to control (Figure 3I).

### Phenotype of Late Osteogenic Differentiation

RUNX2, COL1A1 and OCN are considered important players in the development and maturation of osteogenic processes. RUNX2 expression rises in early osteoblast phenotype, whereas high expression of COL1A1 and OCN are considered hallmarks of late-stage osteoblast differentiation.

**Figure 4:**
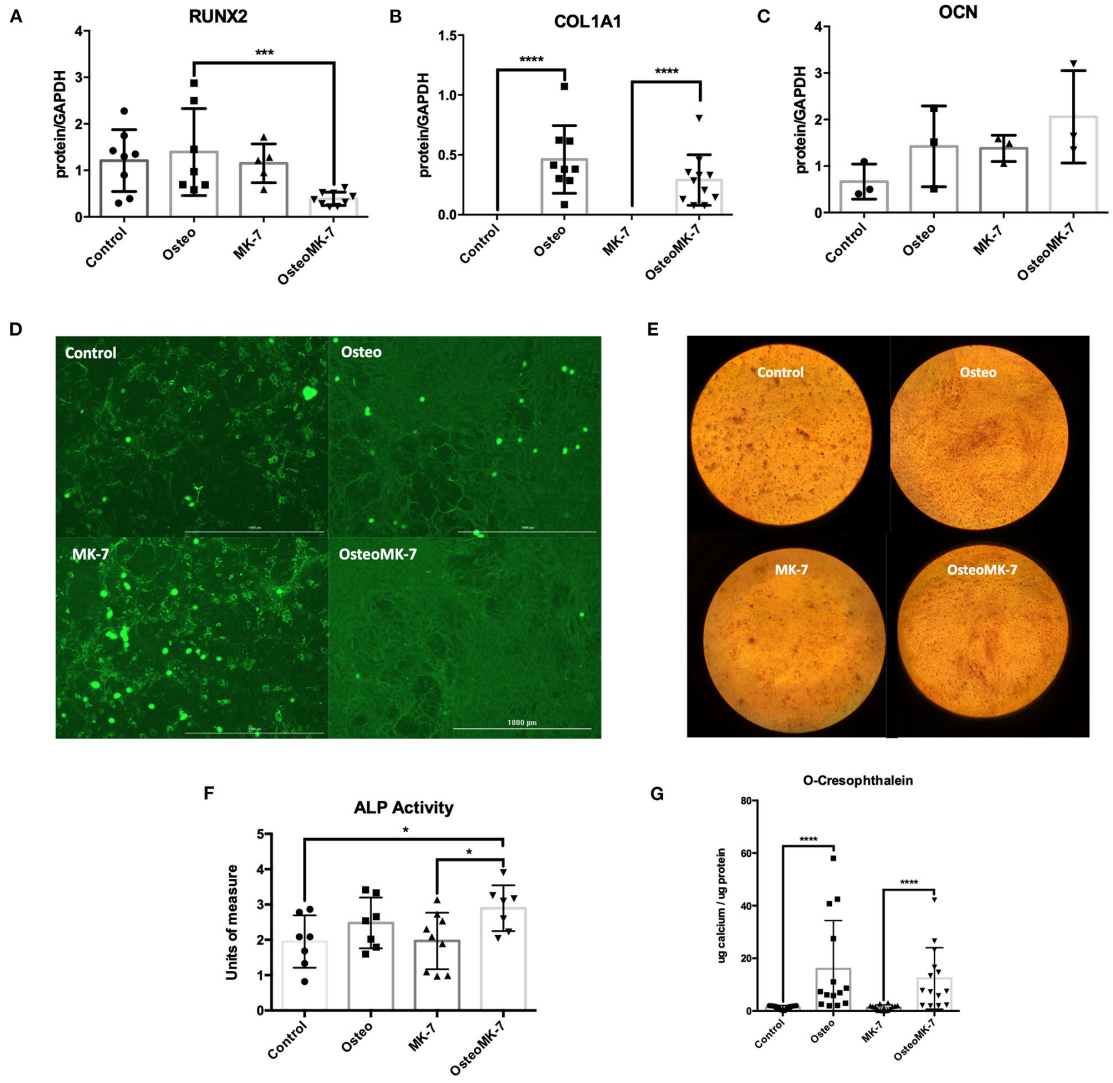
Protein expression at late osteoblast differentiation. Western blotting following 21 days differentiation for RUNX2 shows that following 21 days osteogenic treatment with MK-7 that RUNX2 is significantly downregulated compared to other samples **(A)** (*p* = 0.0003). Collagen 1A1 is upregulated in osteogenic conditions without any difference compared to MK-7 supplementation and OCN has no difference in expression **(B,C)** (*p* ≤ 0.0001). Immunocytochemistry staining for COL1A1 shows that a completely different mode of collagen deposition has occurred following osteogenic treatment, although there are nodules present in the control and control MK-7 treated cells that resemble COL1A1 osteogenic deposition **(D)**. Representative photos of the cells stained with Alizarin Red at day 21 (magnification 10x) **(E)**. At day 21 control, osteogenic and MK-7 treated groups show no difference in ALP levels. Only osteogenic media supplemented with MK-7 was able to induce significant upregulation in ALP activity **(F)** (*p* = 0.0418). Quantification of calcium deposition by o-cresophthalein assay at day 21 **(G)**. *p < 0.05, ***p < 0.001, ****p < 0.0001.

We investigated the cellular phenotype following 21 days of osteogenic differentiation. At the late osteogenic time point, western blot analysis reveals RUNX2 is significantly downregulated under OsteoMK-7 compared to Osteo and both controls (*p* < 0.0001, Figure 4A). Protein expression of COL1A1 reveals a significant upregulation under both Osteo and OsteoMK-7 conditions compared to their respective controls (*p* < 0.001, Figure 4B) but no difference was found between Osteo and OsteoMK-7. Expression of OCN confirm our transcriptional findings, as there is little to no expression of OCN in our cells under any condition (Figure 4C). Immunocytochemistry at day 21 points toward a different mode of COL1A1 expression between osteogenic and control treatments. Although the control samples show that nodules of iMSCs can resemble the osteogenic mode of COL1A1 (Figure 4D), image analysis between Osteo and OsteoMK-7 of fluorescence per cell reveals an upregulation of COL1A1 in OsteoMK-7 compared to Osteo. However, we found this not to be statistically significant (*p* = 0.067, data not shown).

Next we determined the osteogenic phenotype of iMSCs. Alkaline Phosphatase (ALP) activity was significantly upregulated in OsteoMK-7 compared to control and MK-7 (*p* < 0.05, Figure 4F), whereas no statistical difference was found between Osteo and controls. Additionally, both increased AR staining and calcium deposition (o-cresophthalein assay) was observed under osteogenic treatments compared to controls (*p* < 0.0001) for both Osteo and OsteoMK-7 (Figures 4E,G). There was no statistical difference between Osteo and OsteoMK-7 in ALP activity and calcium deposition (Figures 4F,G).

### Effect of MK-7 on Migration, ROS and Cell Cycle Regulator P21

**Figure 5:**
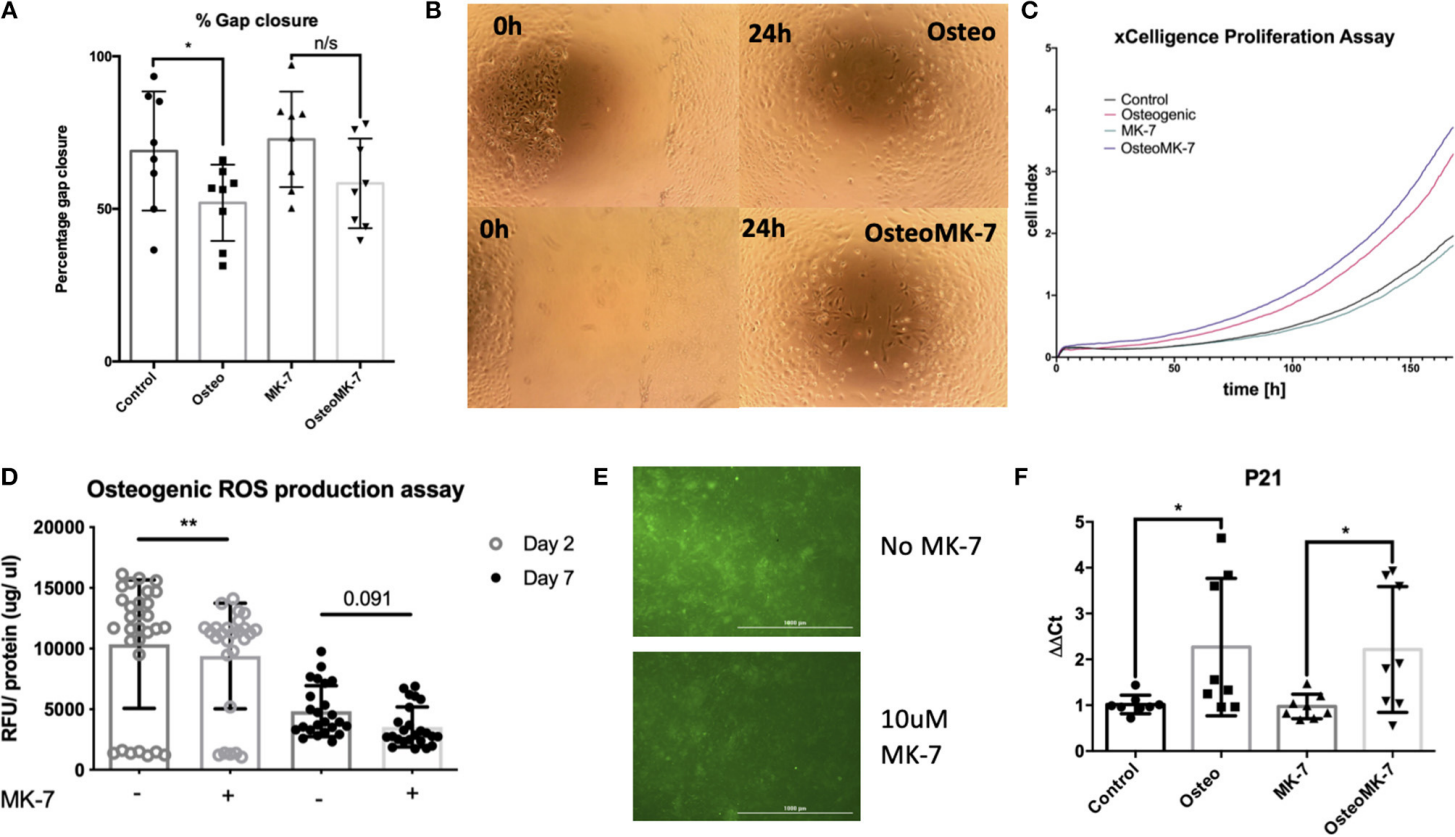
Activity of MK-7 on Migration, ROS and cell cycle regulator P21. Scratch wound assay reveals that MK-7 has the greatest gap closure followed by control, then OsteoMK7 and Osteo last **(A)**. While the difference between osteogenic medium and control alone is statistically significant (*p* = 0.0499), there is less of a difference in gap closure between OsteoMK-7 and MK-7 **(B)**. xCELLigence proliferation assay reveals that OsteoMK-7 has the greatest increases in proliferation over 1 week compared to the other conditions **(C)**. OsteoMK-7 had significantly higher proliferation (*p* < 0.0196) compared to Osteo alone. Further MK-7 supplementation significantly reduces the production of ROS species under osteogenic conditions compared to the osteogenic control **(D,E)** (*p* = 0.0047). Wherein by day 7 of treatment the differences are no statistically significant but remain **(D)** also shown by visual representation of the DCFDA staining between the two different conditions **(E)**. Further at day 2 we note by RT-qPCR analysis, a significant upregulation of cell cycle regulator P21 under both osteogenic conditions compared to controls **(F)** (*p* = 0.0379, *p* = 0.0499). *p < 0.05, **p < 0.01.

Since it is known in bone repair that following fracture and initial hematoma formation, osteoblastic or chondrocytic cells migrate to repopulate the fracture site before mineral deposition occurs (Einhorn and Gerstenfeld, 2015). Therefore, we determined whether cellular migration and proliferation was modulated following Osteo or OsteoMK-7 treatment. For cellular migration we used *in vitro* scratch wound assay. Scratch wound assay demonstrated that MK-7 increases gap closure compared to other conditions (Average closure—control 68.95%, osteo 51.97%, MK7 72.8%, OsteoMK7 58.34%, Figures 5A,B). Further, there was a statistical significance between control and Osteo on gap closure, however between MK-7 and OsteoMK-7 no such observation was present (Figures 5A,B). Next, we investigated the proliferative capacity of cells using the xCELLigence proliferation assay. Following the trend of the findings from scratch wound, both control and MK-7 have the greatest initial proliferation (Figure 5C). However, as the 7-day time frame progresses OsteoMK-7 proliferative capacity increases significantly beyond that of the other conditions, and significantly beyond that of Osteo (*p* < 0.0196). Given that ROS production has been implicated as a driving factor in cellular adhesion and migration, reduced wound healing and osteoporosis (Maggio et al., 2003; Sendur et al., 2009; Schröder, 2013), we investigated ROS species production. Measuring intracellular oxidative stress using DCFDA probe revealed that OsteoMK-7 cells had a significantly reduced level of ROS production compared to osteo at day 2 (*p* < 0.005, Figures 5D,E). Following 1 week of culture, OsteoMK-7 maintains reduced levels of ROS species, although this was found to be no longer statistically significant (*p* < 0.091, Figure 5C). Lastly, given that osteogenic phenotype is a terminal differentiation, cell cycle arrest of osteocytes will provide feedback between ROS production and cell cycle regulator P21. We found P21 expression to be significantly upregulated in osteo and OsteoMK-7 treatments compared to respective controls (p < 0.05, Figure 5F).

### Changes to Dynamics of COL1A1 Expression

**Figure 6:**
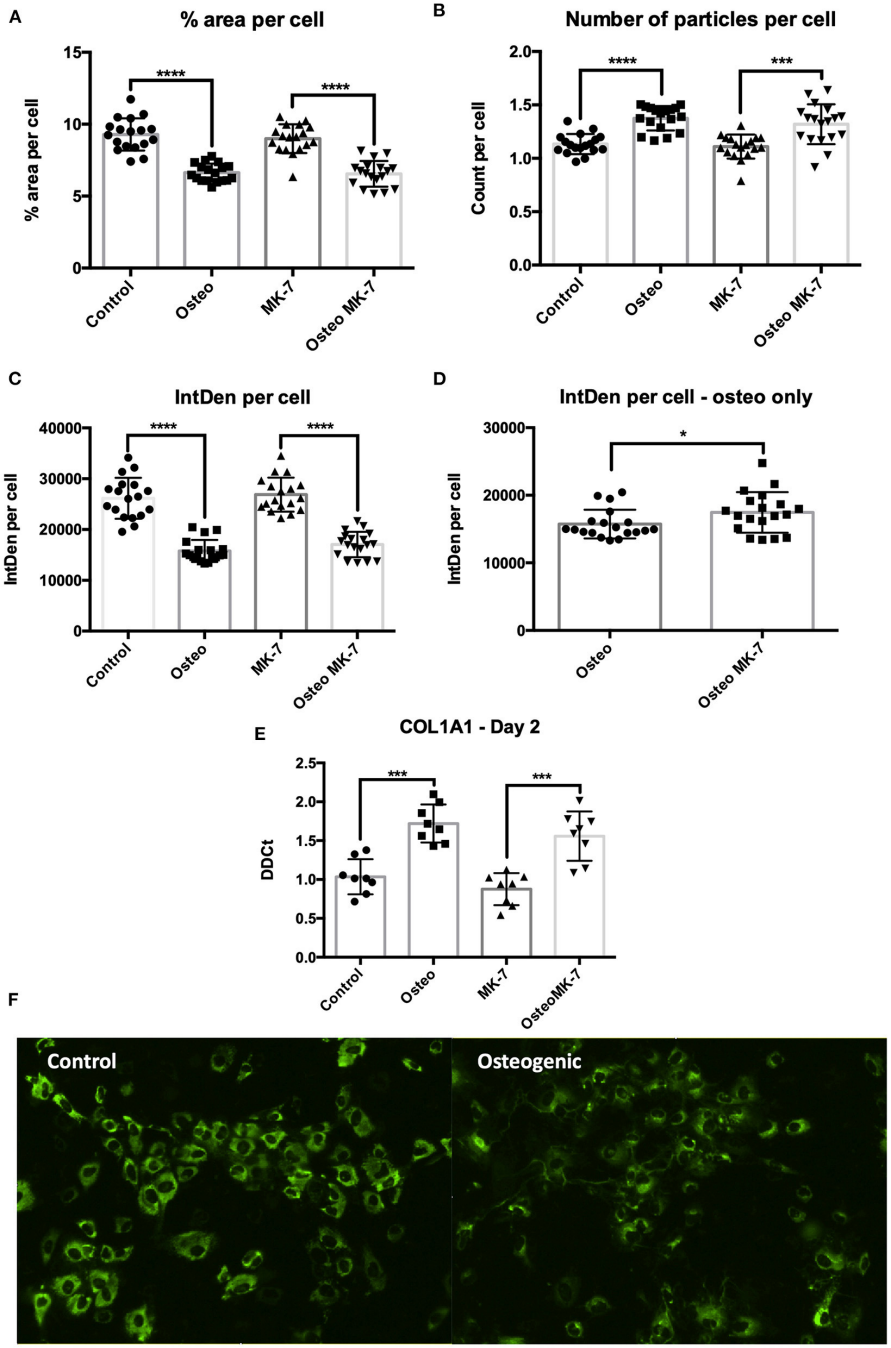
Changes to dynamics of COL1A1 expression. Immunocytochemistry followed by ImageJ analysis reveals that there is a greater surface of COL1A1 expression in control mesenchymal stem cells compared to their respective controls **(A,C)**. The dynamics by which COL1A1 is expressed can be quantified with regards to number of particles expressing COL1A1, wherein there is a significantly greater number of particles, averaging of a smaller size in both osteogenic treatments compared to their respective controls **(B,E)**. When osteogenic treatment is compared to osteogenic treatment with MK-7 supplementation. MK-7 supplementation upregulates the total IntDen (product of area and mean gray values) compared to just osteogenic treatment **(D)**. RT-qPCR confirms significant upregulation of COL1A1 expression at day 2 compared to controls (*p* < 0.05) **(E)**. Representative photos of collagen deposition under control and osteogenic conditions at day 2 **(F)**. *p < 0.05, ***p < 0.001, ****p < 0.0001.

From day 2, immunocytochemistry reveals changes to the mode by which COL1A1 is expressed. Image analysis reveals that not all iMSCs are expressing COL1A1 in any condition at day 2. There is a significantly greater total COL1A1 coverage in control samples at day 2 as quantified by percentage surface area coverage per cell and the product of area per cell IntDen per cell (Figures 6A,C). Similar to that of day 21, we note a differential mode by which COL1A1 is expressed. In control treatments, COL1A1 positive expression appears to be purely cytoplasmic, whereas in both osteogenic treatments, strands or fibers of COL1A1 are detected (Figure 6F). Additionally RT-qPCR confirms a significant (*p* < 0.05) upregulation in COL1A1 expression under osteogenic conditions although no difference between Osteo and OsteoMK-7 was observed (Figure 6E). Using ImageJ analysis, we normalized thresholds equally amongst all images before reducing background, then converting to 8-bit black and white photos which were used for particle analysis and area measurements. This reveals that there were a significantly greater number of particles per cell in osteogenic treatments compared to respective controls (*p*> 0.0001, Figure 6B). Although there was a greater number of particles in osteogenic treatments, we noted no difference in number of particles between Osteo and OsteoMK-7 (Figure 6B). However, when examining the area coverage of these particles (IntDen per cell), we found there to be a significantly greater coverage under OsteoMK-7 compared to Osteo (*p* < 0.05, Figure 6D).

## Discussion

In this study, we derived iMSCs using a simple low-glucose DMEM medium with l-ascorbic acid as previously described (Kang et al., 2015). As this basic medium has no addition of cytokines, growth factors, or compounds we consider it to be cost-efficient. This was sufficient for generation of a population of CD73^+^/CD34^−^ iMSCs. We demonstrated that these cells not only possess the plasticity as expected by primary MSCs, but can both proliferate and maintain this plasticity up to passage 20. We further showed that supplementation of osteogenic differentiation medium with MK-7 increased expression of osteogenic markers at both early and late timepoints of osteoblast differentiation. Additionally, we noted an improved osteoblast phenotype assessed by a variety of assays at early and late time points. The present study shows for the first time that MK-7 can influence the *in vitro* osteogenic differentiation of pluripotent stem cell derived MSCs.

Our first aim was to determine whether reproducibility of the most cost-efficient method for deriving iMSCs from iPSCs was robust. Following 2 weeks of over confluency, two passages on gelatine coated plates (0.1%w/v), and plastic adherence, confirmation of mesenchymal lineage by FACS analysis. We demonstrate iMSCs possess the plasticity as expected *via* the canonical differentiation triad, with an added vasculogenic phenotype.

We next aimed to decipher various stages of osteogenic differentiation. As our iMSC cell line was negative for hematopoietic markers, we are confident that all observations at various timepoints are *via* mesenchymal to osteoblast differentiation (Hutchings et al., 2020). We hypothesize that any observations and understanding derived would be independent of osteoclast involvement in bone formation. Moreover, we interrogated the differentiation trajectory, given basic and clinical science reports on pro-osteogenic properties of MK-7, such as promoting bone formation, bone density and strength, as well as inhibiting bone loss (Sato et al., 2020). Furthermore, vitamin K deficiency is associated with increased fracture rate and decreased bone mass density in a variety of patient cohorts (Tsugawa and Shiraki, 2020). Therefore, we determined whether MK-7 supplementation may further propagate an osteogenic phenotype of iMSCs.

We selected three timepoints based on previous reports to determine insight to early-, mid- and late osteoblast formation (Ciuffreda et al., 2016). At early timepoint, master regulator of osteogenesis, RUNX2 was significantly upregulated in osteogenic medium with MK-7, an observation that hasn't been reported before, and one suggesting that MK-7 might drive early osteogenic differentiation. Additionally, we did not note a difference in expression of BMP2 or OCN at this time point, confirming what has been reported by others (Luu et al., 2007). This suggests to a potentially unknown mechanism by which MK-7 might drive the early stages of osteogenic differentiation from the iMSC intermediate.

At both the mid- and late-osteoblast differentiation stages we observed an upregulation of RUNX2 and COL1A1 as expected, although there is no further benefit of MK-7 activity at this stage. Expression of OCN maintains itself as unaffected by either osteogenic or MK-7 stimulation at these stages. This is contrary to reports that used MC3T3-E1 cell line and primary BM-MSCs wherein an upregulation in expression of OCN has been reported (Ichikawa et al., 2006). It is plausible that OCN expression is different in iPSC derived cells than in cell lines or primary MSCs, and that changes to the carboxylation status of OCN might in part explain why we do not note differences in OCN expression.

It has been reported that RUNX2 is downregulated and possibly even not expressed in mature osteoblasts (Komori, 2020). Our finding corroborates as to a more mature phenotype as protein expression of RUNX2 was significantly downregulated in MK-7 supplemented osteogenic medium. We note little to no protein expression of OCN in any conditions, suggesting that OCN expression is not modulated by MK-7 in osteoblast differentiation. Furthermore, significant upregulation of ALP activity and calcium deposition suggests a more mature osteoblast phenotype. No difference for MK-7 supplementation compared to osteogenic medium was found in calcification propensity, which might be due to the excess calcium ions in cell culture medium present for *in vitro* hydroxyapatite formation.

Collagen is the major collagen constituent of bone. Here, we note a different mode of COL1A1 expression, with strands or fibers of collagen present in totality over the osteogenic differentiation. In controls, COL1A1 appear to be present in nodules wherein a cluster of iMSCs have possibly spontaneously osteogenically differentiated (Alami et al., 2016). We show a greater surface area of osteogenic differentiation with MK-7 although not statistically significant. Despite there being no increased *in vitro* calcification on adding MK-7, our findings suggest a more mature osteogenic cellular phenotype by downregulation of RUNX2, increased collagen deposition, and increased ALP activity.

Since upregulation of RUNX2 is indicative of an early pro-osteogenic event by MK-7 activity, we investigated the potential of wound healing. MK-7 supplementation increased gap closure and increased proliferation compared to osteogenic medium alone. It is widely accepted that oxidative stress accelerates the rate of bone loss and is one of the risk and pathogenic factors in osteoporosis (Sánchez-Rodríguez et al., 2007; Manolagas and Parfitt, 2010; Bonaccorsi et al., 2018). Our group has previously noted increases in production of ROS species to be associated with ectopic vascular calcification *in vitro* (Furmanik et al., 2020). Intriguingly, ROS in osteogenic iMSCs were significantly downregulated by MK-7 treatment, which is in concordance with literature wherein antioxidants decreased oxidative stress in osteoblasts (Rao and Rao, 2015). This suggest that MK-7 counteracts oxidative stress in developing bone *in vitro* and should be further explored for its antioxidative properties.

As we note increases in cell number with MK-7 supplementation, nodularity of conditions inducing osteogenic events, and given that ossification is considered a terminal differentiation process, we analyzed expression of cell cycle regulator P21. Increases in P21 expression are typically associated with transcriptional regulation of cell cycle arrest (Gartel and Radhakrishnan, 2005). In both osteogenic conditions P21 is significantly upregulated, however increases in proliferation under OsteoMK-7 has been noted. This suggests there might be an overriding regulatory mechanism induced by MK-7. Our data suggests that MK-7 decreases ROS production while increasing cellular migration, proliferation, and potentially overriding cell cycle regulation by P21 during osteogenesis, providing novel insights into the activity of MK-7 on osteogenesis.

Differences in collagen expression amongst samples were apparent and detectable from as early as day 2. Under osteogenic conditions many of the cells appear to have COL1A1 positive fibers, an observation that is completely absent in controls. Furthermore, at this time point the dynamics by which extracellular COL1A1 is expressed is different in osteogenic treatment and MK-7 increases COL1A1. Combined, our data reveal novel insights into the mesenchymal osteogenic differentiation as well evidence for use and efficacy of MK-7 in promoting an osteogenic cellular phenotype.

We did not find OCN expression modulated at any time point. Additionally, in the early timepoint we note no change to BMP-2 activity, which contradicts with literature (Luu et al., 2007). This suggests to additional non-canonical roles of MK-7 in pluripotent mesenchymal differentiation, that remains elusive from our findings. Furthermore, we purposefully chose to use iMSCs between passages 10-20 to determine whether previously noted increased passaging capabilities were also reproducible in our model. Given that primary mesenchymal stem cells typically senesce by passage 8, this further advocates for use of iMSCs as a more practical and scalable cellular resource (Mareschi et al., 2006).

We note that there are particular limitations to this study. Firstly, our findings have only been validated *in vitro*. It is entirely plausible that the benefit to adding MK-7 is a cell culture phenomenon wherein decreasing cellular stress under high phosphate conditions, for increased cellular proliferation and differentiation. Additionally, we note no differences in calcium deposition, despite increased ALP activity. As we did not vary calcium concentrations this could justify why we do not find differences in calcification with MK-7 supplementation.

This study provides a framework for future studies, both *in vitro* and *in vivo* for the role of MK-7 in bone formation and fracture healing. This corroborates observations, from longitudinal and interventional trials on increased bone mineral density following either vitamin K2 supplementation (Sato et al., 2020) or an increased amount of vitamin K2 in the diet (Tsugawa and Shiraki, 2020). Furthermore, the role of VKDPs in iPSC derived products has not been widely explored. Additionally, our data suggest non-canonical function of VKDPs in early differentiation events, that can be modeled using iPSCs. Lastly, the increasing osteogenic cellular phenotype might prove beneficial toward development of tissue engineering solutions such as *ex vivo* bone grafts. In turn, development of biological products over ceramic or metallic based solutions could be more advantageous over artificial products. There is much we do not know about differentiation processes and extrahepatic activity vitamin K2 analogous. This paper provides a first account into several mechanisms of osteogenesis from iPSCs and the influence of MK-7 on osteogenic processes.

## Data Availability Statement

The raw data supporting the conclusions of this article will be made available by the authors, without undue reservation.

## Author Contributions

AA, GW, NR, KC-N, and LS contributed to writing this manuscript. FF, HS, and KC-N generated and characterized the iPSC line. AA, GW, NR, and FF performed the experiments. NR and KC-N carefully read and contributed to writing the manuscript. AA, GW, and LS drafted and edited the manuscript. AA and GW together designed the experiments and performed the analysis. AA, KC-N, and LS conceived the study. AA and LS were responsible for the final version of the manuscript. All authors contributed to the article and approved the submitted version.

## Conflict of Interest

LS reports financial support from Boehringer Ingelheim, Daichi Sankyo, Bayer, Nattopharma, and Immunodiagnostic systems (IDS), outside the submitted work. GW was employed by Nattopharma. The remaining authors declare that the research was conducted in the absence of any commercial or financial relationships that could be construed as a potential conflict of interest.
