# Supplementary material for: Menaquinone-7 Supplementation Improves Osteogenesis in Pluripotent Stem Cell Derived Mesenchymal Stem Cells
Source: Front Cell Dev Biol. 2021 Jan 28;8:618760. doi: 10.3389/fcell.2020.618760 (PMC7876270; doi:10.3389/fcell.2020.618760)
Supplement: Supplementary file 1 [file Table_1.DOCX]

**Table 1 – qPCR primers**

| Gene |  |
| --- | --- |
| RUNX2 | QIAGEN, QT00020517 |
| BMP-2 | QIAGEN, QT00012544 |
| OCN | F: GGCAGCGAGGTAGTGAAGAG  R: CGATAGGCCTCCTGAAAGC |
| COL1A1 | F: TGTGGCCCAGAAGAACTGGTACAT  R: ACTGGAATCCATCGGTCATGCTCT |
| GAPDH | F: AACGGATTTGGTCGTATTGGGC  R: CTTGACGGTGCCATGGAATTTG |
| P21 | F: GCAGACCAGCATGACAGATTTC  R: GCGGATTAGGGCTTCCTCTT |
